# Bioprospecting the American Alligator (*Alligator mississippiensis*) Host Defense Peptidome

**DOI:** 10.1371/journal.pone.0117394

**Published:** 2015-02-11

**Authors:** Barney M. Bishop, Melanie L. Juba, Megan C. Devine, Stephanie M. Barksdale, Carlos Alberto Rodriguez, Myung C. Chung, Paul S. Russo, Kent A. Vliet, Joel M. Schnur, Monique L. van Hoek

**Affiliations:** 1 Department of Chemistry and Biochemistry, George Mason University, Fairfax, Virginia, United States of America; 2 School of Systems Biology, George Mason University, Manassas, Virginia, United States of America; 3 Center for Applied Proteomics and Molecular Medicine, George Mason University, Manassas, Virginia, United States of America; 4 Department of Biology, University of Florida, Gainesville, Florida, United States of America; 5 College of Science, George Mason University, Fairfax, Virginia, United States of America; 6 National Center for Biodefense and Infectious Diseases, George Mason University, Manassas, Virginia, United States of America; University Hospital Schleswig-Holstein, Campus Kiel, GERMANY

## Abstract

Cationic antimicrobial peptides and their therapeutic potential have garnered growing interest because of the proliferation of bacterial resistance. However, the discovery of new antimicrobial peptides from animals has proven challenging due to the limitations associated with conventional biochemical purification and difficulties in predicting active peptides from genomic sequences, if known. As an example, no antimicrobial peptides have been identified from the American alligator, *Alligator mississippiensis*, although their serum is antimicrobial. We have developed a novel approach for the discovery of new antimicrobial peptides from these animals, one that capitalizes on their fundamental and conserved physico-chemical properties. This sample-agnostic process employs custom-made functionalized hydrogel microparticles to harvest cationic peptides from biological samples, followed by *de novo* sequencing of captured peptides, eliminating the need to isolate individual peptides. After evaluation of the peptide sequences using a combination of rational and web-based bioinformatic analyses, forty-five potential antimicrobial peptides were identified, and eight of these peptides were selected to be chemically synthesized and evaluated. The successful identification of multiple novel peptides, exhibiting antibacterial properties, from *Alligator mississippiensis* plasma demonstrates the potential of this innovative discovery process in identifying potential new host defense peptides.

## Introduction

There has been a growing interest in cationic antimicrobial peptides (CAMPs) as a potential source of new therapeutics with which to address the growing problem of bacterial antibiotic resistance [1, 2]. Nature provides a prescreened library of peptides that has been selected over millions of years of evolution for their ability to defend against infection under physiological conditions. The American alligator (*Alligator mississippiensis*) and other crocodilians are evolutionarily ancient animals whose plasma and leukocyte extracts have been shown to exhibit antimicrobial activity [3–5]. This antimicrobial potency may be attributable at least in part to the presence of CAMPs in the alligator plasma and extracts. Cationic antimicrobial peptides have been shown to be capable of exerting significant antibacterial effects, and they figure prominently in the innate immunity of vertebrates and other higher organisms. Despite the interest in the American alligator and the antimicrobial peptides that they may produce, no CAMPs have been identified from their blood or tissues to date [3, 6].

The discovery and identification of novel CAMPs from animals has proven challenging using conventional proteomics tools. Methods used to fractionate and isolate peptides are labor-intensive, can result in sample and activity loss, and are unable to detect low-abundance peptides. To address these limitations, prior efforts to identify crocodilian antimicrobial peptides have resorted to using very large sample volumes [5, 7], which can be problematic if the animals are endangered or sample size is limiting. Further complicating matters, the high sequence and structural diversity of CAMPs presents an impediment to traditional bottom-up proteomics mass spectrometry methods, which employ proteolytic digestion and database searches to facilitate peptide sequence determination. Subjecting samples to proteolytic digestion in this manner destroys information regarding the original native, intact peptide sequences. To overcome these challenges, we have employed a multidisciplinary strategy that draws from protein biophysics, peptide chemistry, nanomaterials, advanced mass spectrometry techniques, and microbiology.

**Fig 1:**
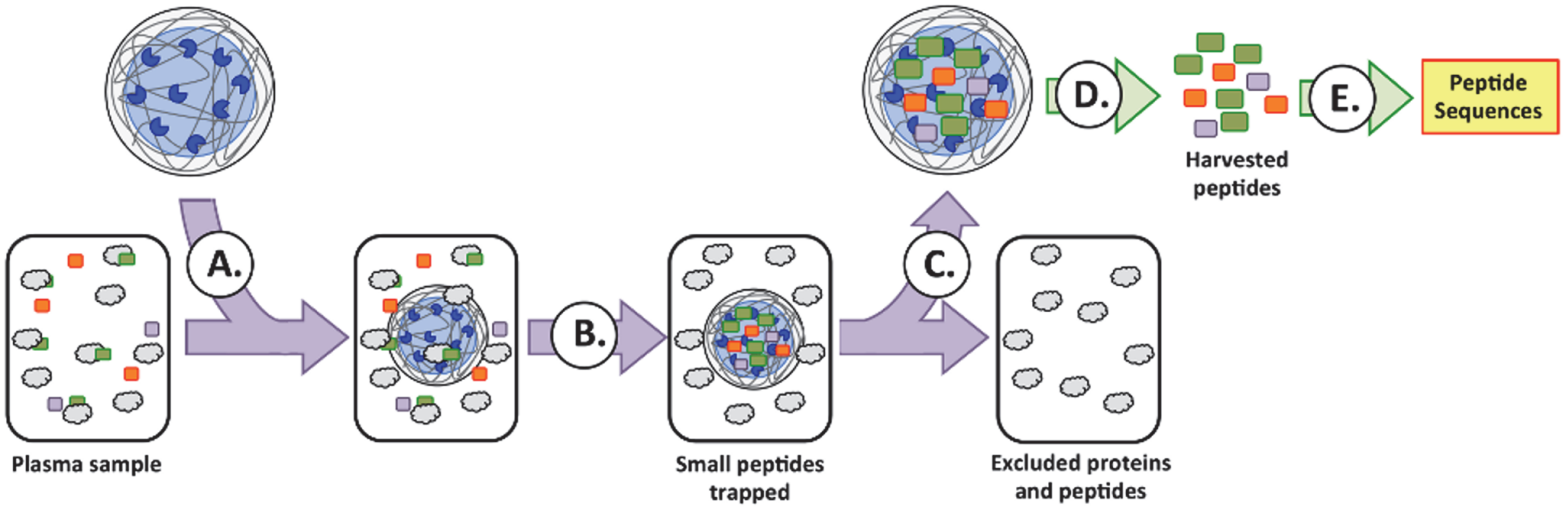
Bioprospecting Approach to CAMP discovery. (A) Hydrogel microparticles are introduced into the plasma sample. (B) The particles capture small cationic peptides which are present in the sample, while excluding high molecular weight proteins. (C) The particles are then recovered, (D) captured low molecular weight peptides are eluted from the particles and (E) analyzed by high-resolution MS/MS.

We report here the development of a new bioprospecting particle-assisted proteomics approach to antimicrobial peptide discovery, which builds upon recent advances in proteomics and biomarker discovery [8, 9]. It utilizes a novel approach for extracting peptides, including CAMPs, from very small sample volumes (e.g. 100µl) followed by analysis of the harvested peptides using advanced middle-down mass spectrometry techniques and *de novo* peptide sequencing to identify CAMPs that may be present (Fig. 1). We have applied this process to the discovery of novel CAMPs from plasma from the American alligator. It employs custom-made functionalized hydrogel microparticles to harvest CAMPs and CAMP-like peptides in their native form from biological samples, agnostic to source, based on their physico-chemical properties. Mass spectral analysis of the harvested intact peptides using an Orbitrap Elite mass spectrometer equipped with electron transfer dissociation (ETD) is used to determine their sequences in a *de novo* manner. The sequences are compared to available genomic and proteomic information in order to confirm, complete and correct the *de novo* peptide sequences. Furthermore, all sequences are ultimately manually verified, especially those for which no genomic information is available. Two selection criteria are applied to down-select peptides for further testing. In one approach, potential CAMPs are identified from the peptide sequences using web-based prediction tools (*CAMP* database, *AntiBP2* and *APD2*) [10–12]. In the other approach, prospective CAMPs are selected through rational analysis of the peptide sequences, focusing primarily on size, charge, and sequence similarity with other potential CAMPs. In our analysis of alligator plasma, we identified forty-five potential CAMPs. In this process, the particle harvest is used for peptide discovery, not purification. For further testing, identified cationic peptides must be synthesized and their antimicrobial activities determined. In order to provide an initial assessment of the rational and web-based CAMP prediction methods, eight CAMP candidates were synthesized and evaluated for activity. Four of the eight peptides were selected because they had physico-chemical properties consistent with known CAMPS, specifically being highly cationic with nominal charges ranging from +4 to +5 (APOC1_64–88_, APOC1_67–88_, A1P_394–428_, ASAP130LP). The next two peptides were chosen because the predictive algorithms indicated a high probability that they would be antimicrobial despite having lower net cationic charge of +2 and +3 (AVTG2LP and NOTS_17–38_). The remaining two peptides were selected because they exhibited overlap between both selection criteria, having both positive CAMP prediction scores and good physico-chemical properties (FGG_398–413_ and FGG_401–413_). Evaluation of these eight peptides led to the identification of five novel alligator peptides that demonstrate antimicrobial activity, APOC1_64–88_, APOC1_67–88_, A1P_394–428_, FGG_398–413_ and FGG_401–413_.

## Results

The first step in the bioprospecting process used to identify novel alligator CAMPs employed hydrogel microparticles based on cross-linked N-isopropylacrylamide copolymer frameworks, which are central to the CAMP discovery process [8, 9]. Harvesting was performed using a 50:50 combination of two types of particles, one incorporating acrylic acid as its affinity bait and the other combining acrylic acid and 2-acrylamido-2-methyl-propanesulfonic acid as baits. These particles enable multidimensional separation of targeted peptides from other proteins and peptides present in the samples. Negatively charged acidic groups, such as carboxylic acids and sulfonic acids, provide affinity baits for the capture of cationic peptides and proteins. At the same time, the cross-linking of the polymer scaffold excludes larger peptides and proteins, while allowing low molecular weight peptides access to affinity baits residing in the particle interior. Thus, the particles simultaneously combine elements of cation exchange and size-exclusion chromatography when capturing peptides and proteins from complex biological samples, favoring peptides with physico-chemical properties similar to those of CAMPs. Additionally, hydrogel particles have been shown to protect captured labile biomolecules from degradation during the harvesting process [9].

**Fig 2:**
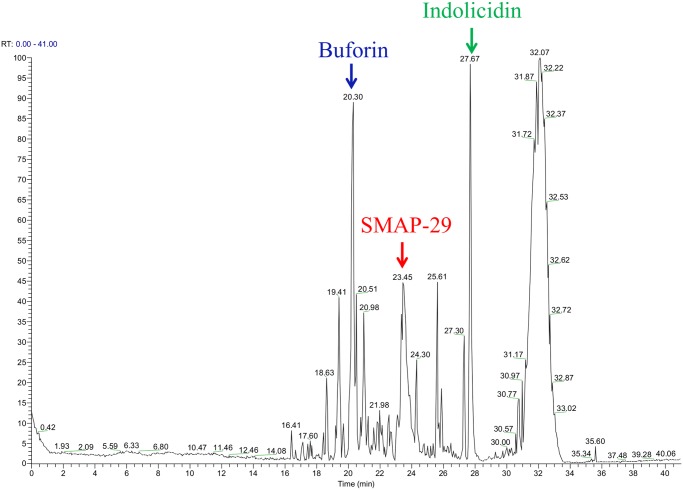
Mass Spectral Verification of CAMP Harvesting by Hydrogel Particles. Mass spectra of particle eluents following harvesting from of American alligator plasma spiked with 3 known CAMPs (Buforin, SMAP-29 and Indolicidin). The CAMP peaks in the spectra are identified and labeled with their corresponding identity.

In our first experiment, we sought to establish that the particles harvested known CAMPs from alligator plasma. Due to the limited available information regarding the CAMPs that may be present in alligator plasma, the ability of the hydrogel particle combination to capture CAMPs from 100 µL of commercial alligator plasma was evaluated using a mixture of known peptides (60 pmol each) representing different CAMP classes: buforin (histone-derived), SMAP-29 (helical), and indolicidin (linear Trp/Arg/Pro-rich). The model-peptide sample was diluted into an aqueous suspension of hydrogel particles, and the harvest mixture was then incubated for 18 hours at room temperature. The particles were recovered by centrifugation and washed to remove unbound, excluded peptides and proteins. Captured peptides were eluted from the washed particles and analyzed by mass spectrometry, which revealed that the particles had effectively harvested the model CAMPs from the plasma along with alligator plasma peptides (Fig. 2). Buforin harvesting was confirmed by the presence of the (M+4H)^+4^ ion at 609.4 m/z, the (M+5H)^+5^ ion at 487.7 m/z and the (M+6H)^+6^ ion at 406.6 m/z. SMAP-29 capture was identified by the (M+5H)^+5^, (M+6H)^+6^ and (M+7H)^+7^ at 651.8 m/z, 543.3 m/z and 465.9 m/z, respectively. The harvesting of indolicidin was verified by the presence of the (M+3H)^+3^ ion at 636.0 m/z and the (M+4H)^+4^ ion at 477.3 m/z, thus demonstrating the ability of the particles to capture known CAMPs and CAMP-like peptides from plasma.

With the ability of the hydrogel particles to capture known CAMPs from plasma established, the harvesting process was then applied to stimulated alligator plasma for the identification of novel crocodilian CAMPs. Prior to harvesting, alligator blood was treated with ionomycin to stimulate peptide release into the plasma from heterophils. Ionomycin, which is a calcium ionophore, has been demonstrated to trigger the release of hCAP18 (the human cathelicidin LL-37 precursor) from neutrophil granules [13]. Following stimulation, the plasma and cells were separated and a broad-spectrum protease inhibitor cocktail was added in order to prevent proteolysis of the released peptides. Particle harvesting was then performed from 100 µL of stimulated plasma. The particles were collected and washed, with the captured peptides remaining trapped in the interior of the microparticles. The harvested peptides were eluted and de-salted for mass spectrometry.

**Fig 3:**
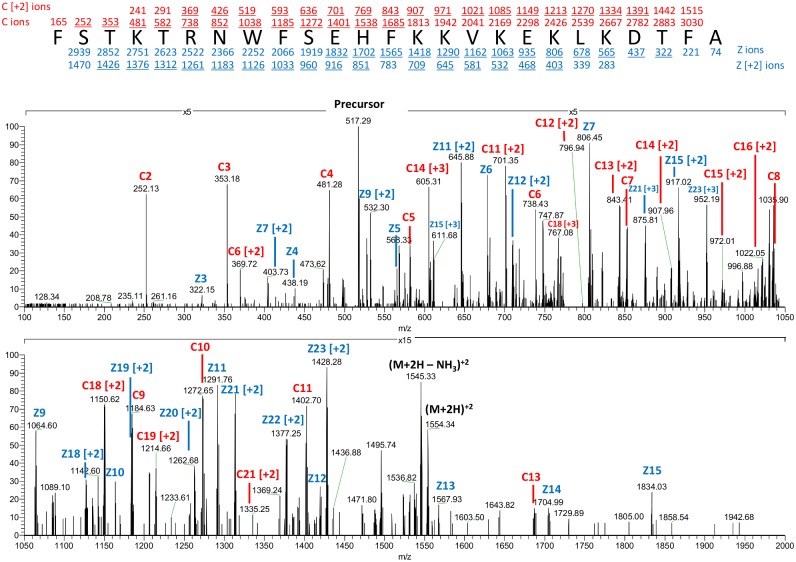
ETD Mass Spectrum for APOC1_64–88_. ETD mass spectrum recorded for the 25-residue peptide on the (M+6H)^+6^ ion at m/z 517.95 (MW 3101.65 Da). Observed singly, doubly and triply charged c (red) and z (blue) ions are indicated on the peptide sequence and are labeled in the spectrum. (Ions present in the spectrum are underlined).

The second step in the bioprospecting process is the identification and sequencing of potential CAMPs. The sequences of captured native intact peptides, including potential CAMPs, were elucidated using an Orbitrap Elite mass spectrometer equipped with ETD fragmentation, which has been shown to be ideally suited for fragmenting large, highly charged peptides [14, 15]. When combined with the high sensitivity, resolution and mass accuracy of the Orbitrap, ETD can be used for the *de novo* sequencing of full-length functional peptides. Here, ETD spectra were analyzed by PEAKS software to sequence peptides in a *de novo* manner. PEAKS then uses sequence tags from the *de novo* sequences to search both the American alligator EST database and transcriptome database [16]. However, not all of the *de novo* peptide sequences are found in this database. Peptides of interest, both those that are confirmed from the database and those that have no database equivalent, were all manually verified. To illustrate how peptide sequences can be derived *de novo* from ETD mass spectra, a representative spectrum is presented in Fig. 3.

The bioprospecting CAMP-discovery process, coupling hydrogel particle harvesting of peptides with advanced mass spectrometry techniques, was applied to 100 µL of stimulated alligator plasma. This resulted in the capture of thousands of peptides, all less than 15 kDa in weight, with tandem MS/MS analysis by ETD leading to the sequencing of 568 peptides. A combination of web-based CAMP prediction algorithms and rational peptide sequence analysis based on known CAMP physico-chemical properties are used to identify probable CAMP sequences for synthesis and testing.

Several bioinformatic tools have been developed in an attempt to predict novel CAMPs [10–12]. All 568 sequences were initially submitted for evaluation using three web-based CAMP prediction tools: *CAMP* database, *AntiBP2* and *APD2*. *CAMP* database uses three different algorithms, Support Vector Machine (SVM), Random Forest (RF) and Discriminate Analysis (DA), to predict likelihood of antimicrobial activity [10]. *AntiBP2* uses SVM to determine CAMP potential [11]. The third program, *APD2*, makes a qualitative determination based on the probability of the input sequence being antimicrobial in comparison to the sequences of known antimicrobial peptides in its database [12]. The rational analysis approach focuses on physico-chemical properties that can be calculated based on the peptide sequences, such as molecular weight, length, charge at physiological pH, peptide isoelectric point, and hydrophobicity. Only sequences corresponding to peptides with molecular weights of less than 5.5 kDa were considered, because this is consistent with the molecular weights of the majority of known vertebrate CAMPs. Sequences with charge of +4 or higher were considered, because high cationic character is believed to be linked with activity. Since peptide isoelectric point and hydrophobicity varies widely amongst known CAMPs, these properties were not used as a primary consideration in determining antimicrobial potential. These two approaches to CAMP identification (CAMP-prediction algorithms and rational analysis) are not mutually exclusive, and were both used in order to capture the greatest number of credible peptide sequences that have the potential for antimicrobial activity. Peptide sequences that showed positive CAMP prediction scores or exhibited physico-chemical properties associated with known CAMPs were added to our list of potential CAMPs. The lists of potential CAMPs generated by the two prediction approaches were consolidated and duplicates eliminated.

Based on these prediction methods, forty-five of the 568 peptide sequences were identified as potential CAMPs. From this list, eight peptides (APOC1_64–88_, APOC1_67–88_, A1P_394–428_, FGG_398–413_, FGG_401–413_, AVTG2LP, ASAP130LP and NOTS_17–38_) were selected for further study and synthesized in order to evaluate their antimicrobial activities (Table 1). The peptides APOC1_64–88_, APOC1_67–88_, A1P_394–428_, and ASAP130LP were selected because of their relatively high theoretical positive charges at physiological pH (ranging from +4 to +5), while AVTG2LP and NOTS_17–38_ were selected because the prediction algorithms generally (4 out of 5) agreed that they were likely CAMPs. The two peptides, FGG_398–413_ and FGG_401–413_, were selected due to the overlap exhibited between both prediction methods.

**Table 1:** Sequences and Physico-chemical Properties of Novel Alligator CAMPs.

**Peptide**	**Sequence**	**Length (res)**	**Molecular Weight (Da)**	**Net Charge**	**pI**	**Hydrophobicity**	**Parent Protein**
APOC1_64–88_	FSTKTRNWFSEHFKKVKEKLKDTFA	25	3101.65	4	10.0	-1.2	Apolipoprotien C1
APOC1_67–88_	KTRNWFSEHFKKVKEKLKDTFA	22	2766.49	4	10.0	-1.4	Apolipoprotien C1
FGG_398–413_	YSLKKTSMKIIPFTRL	16	1925.12	4	10.5	-0.050	Fibrinogin
FGG_401–413_	KKTSMKIIPFTRL	13	1561.94	4	11.3	-0.19	Fibrinogin
A1P_394–428_	PPPVIKFNRPFLMWIVERDTRSILFMGKIVNPKAP	35	4106.28	4	11.0	0.020	Alpha-1-antiproteinase
AVTG2LP*	LQTKLKKLLGLESVF	15	1716.06	2	9.70	0.36	Vitellogenin-2
ASAP130LP*	PPGASPRKKPRKQ	13	1445.85	5	12.0	-2.3	Sin3A-Associated Protein, 130 kDa
NOTS_17–38_	VERIPLVRFKSIKKQLHERGDL	22	2660.56	3	10.3	-0.62	Nothepsin

The physico-chemical properties for eight novel alligator CAMPs identified via the process. The peptide name is determined based on the parent protein with the amino acid sequence numbers in the subscript.

* These peptides were *de novo* identified.

The third step in the CAMP-discovery process is the evaluation of the antimicrobial effectiveness of the newly identified peptides. The eight synthetic peptides were tested against a panel of Gram-positive and Gram-negative bacteria. These bacteria include *Bacillus cereus*, *Staphylococcus aureus*, *Escherichia coli*, and *Pseudomonas aeruginosa*. Antimicrobial assays designed to determine the half-maximal effective concentrations (EC_50_) of the peptides were performed using a high-throughput assay based on resazurin as a reporter for cell viability (Table 2) [17–24]. When incubated with live metabolically-active cells, the non-fluorescent blue dye resazurin is converted to the highly fluorescent pink resorufin, as a result of reduction by the cells. While bacteria can reduce resazurin via multiple metabolic processes, the rate of conversion and the corresponding increase in fluorescence are directly proportional to the number of living cells in the sample [19–21, 25]. Thus, fluorometric detection of the rate of resazurin conversion to resorufin at 530_ex_/590_em_ allows for quantification of bacterial survival following exposure to antibacterial compounds, such as CAMPs [17]. It has been confirmed that the time that bacterial cultures require to achieve specified fluorescence intensities correlates inversely to the initial bacterial concentration, and the results obtained using resazurin-based assays are comparable to those determined using classical dilution-plating assays for evaluating bacterial viability [17, 18, 23, 24].

**Table 2:** Antibacterial Performance Data for Alligator CAMPs.

**Peptide**	***E. coli***		***B. cereus***		***P. aeruginosa***		***S. aureus***	
	**EC_50_ (µM)**	**95% CI**	**EC_50_ (µM)**	**95% CI**	**EC_50_ (µM)**	**95% CI**	**EC_50_ (µM)**	**95% CI**
LL-37	0.00821	0.00591 to 0.0113	0.0287	0.0242 to 0.0341	0.525	0.446 to 0.615	0.552	0.383 to 0.797
APOC1_64–88_	0.192	0.129 to 0.284	0.245	0.223 to 0.269	1.41	0.906 to 2.23	9.66	7.69 to 12.2
APOC1_67–88_	0.151	0.0716 to 0.319	0.210	0.181 to 0.244	0.948	0.706 to 1.27	7.08	5.39 to 9.29
FGG_398–413_	0.332	0.162 to 0.678	9.35	7.75 to 11.3	7.02	5.30 to 9.23	2.84	1.86 to 4.33
FGG_401–413_	0.245	0.150 to 0.360	18.7	wide	11.1	9.30 to 13.4	31.8	18.7 to 54.0
A1P_394–428_	0.0986	0.0478 to 0.203	0.770	0.257 to 2.31	4.35	3.74 to 5.04	1.36	0.925 to 1.99
AVTG2LP	NA	NA	NA	NA	NA	NA	NA	NA
ASAP130LP	NA	NA	NA	NA	NA	NA	101	wide
NOTS_17–38_	NA	NA	NA	NA	NA	NA	198	wide

Antibacterial activities against *E. coli, B. cereus, P. aeruginosa* and *S. aureus* are expressed in terms of EC_50_ (µM) values with corresponding 95% confidence interval (CI) range. The human CAMP LL-37 is used as a standard for assessing antibacterial performance [30, 31]. **NA** = no significant activity at the highest peptide concentration tested against the listed bacteria.

**Fig 4:**
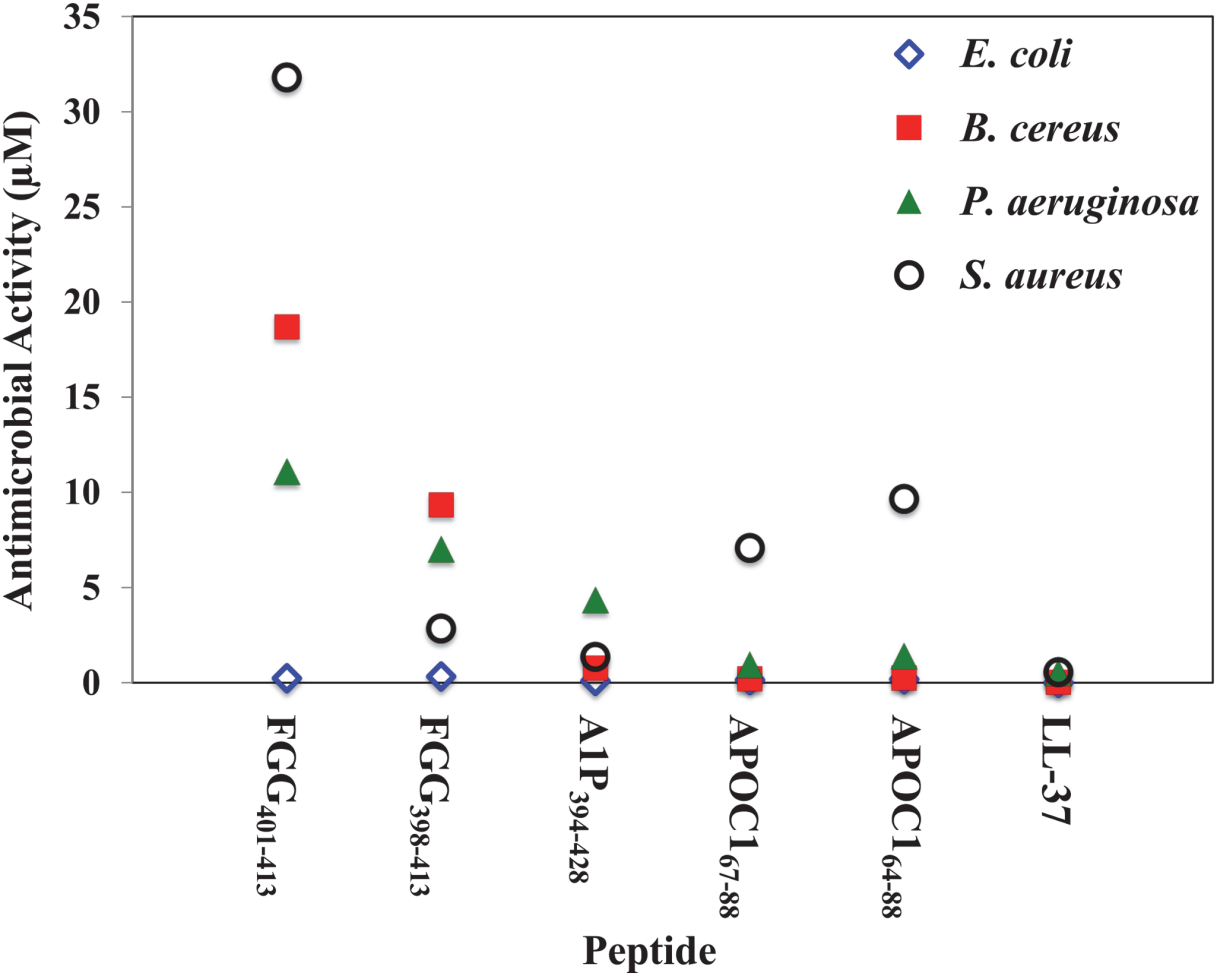
Potencies of LL-37 and Five Novel Alligator CAMPs. Comparison of the antibacterial effectiveness of each peptide against *E. coli, B. cereus, P. aeruginosa* and *S. aureus*, expressed in terms of EC_50_ (µM) values. Bacterial survival results generated for each CAMP are fit to a variable-slope sigmoidal regression model to reveal bacterial survival curves to ascertain EC_50_ values.

Of the eight synthesized peptides, five showed significant antibacterial activity against one or more of the bacteria in the panel, based on EC_50_ values (Fig. 4): APOC1_64–88_, APOC1_67–88_, A1P_394–428_, FGG_398–413_ and FGG_401–413_. Based on CAMP prediction algorithms, NOTS_17–38_ and AVTG2LP were predicted likely to be effective antimicrobial peptides (Table 3). However, neither of these peptides exhibited significant antibacterial activity against the panel of bacteria (Table 2), revealing limitations in the utility of currently available CAMP prediction models [10–12]. Additionally, the peptide ASAP130LP, which appeared to have physico-chemical properties consistent with known CAMPs, also proved ineffective against the panel of bacteria tested.

**Table 3:** CAMP prediction results.

**Peptide**	***CAMP* Prediction Score**			***AnitBP2* Prediction Score SVM**	***APD2* Prediction Probability Qualitative**
	**SVM**	**RF**	**DA**		
APOC1_64–88_	0.894:Non-AMP	0.728:Non-AMP	0.667:Non-AMP	-0.210:Non-AMP	+
APOC1_67–88_	0.598:Non-AMP	0.692:Non-AMP	0.352:Non-AMP	-0.052:Non-AMP	+
FGG_398–413_	0.508:AMP	0.656:Non-AMP	-0.384:AMP	-0.172:Non-AMP	-
FGG_401–413_	0.732:AMP	0.514:AMP	-1.23:AMP	ND*	-
A1P_394–428_	0.935:Non-AMP	0.838:Non-AMP	0.363:Non-AMP	-0.241:Non-AMP	+
AVTG2LP	0.821:AMP	0.386:Non-AMP	0.877:AMP	0.223:AMP	+
ASAP130LP	0.157:Non-AMP	0.445:Non-AMP	0.077:Non-AMP	ND*	+
NOTS_17–38_	0.757:AMP	0.600:AMP	-0.165:Non-AMP	0.618:AMP	+

Using 3 different web-based CAMP prediction applications (*CAMP database, AntiBP2 and APD2*) each peptide was scored and given a prediction of whether it would have antimicrobial activity (AMP) or not (Non-AMP).

* *AntiBP2* requires the sequence length of ≥ 15 amino acids for a prediction score.

*CAMP* database: http://www.camp.bicnirrh.res.in/.

*AntiBP2:* http://www.imtech.res.in/raghava/antibp2/.

*APD2:* http://aps.unmc.edu/AP/prediction/prediction_main.php.

APOC1_64–88_ (25aa) and APOC1_67–88_ (22aa) are a nested pair of peptide fragments of apolipoprotein C1 and share a nominal net charge of +4 at physiological pH. Apolipoprotein C1 is generally associated with lipid metabolism, but it has recently been suggested that the protein may also play a role in inflammation [26]. APOC1_64–88_ and APOC1_67–88_ exerted significant antimicrobial activity against *E. coli*, *P. aeruginosa* and *B. cereus*, but were not as effective against *S. aureus*. The longer peptide APOC1_64–88_ exhibited antimicrobial EC_50_ values of 0.192 µM and 1.41 µM against the Gram-negative bacteria *E. coli* and *P. aeruginosa*, respectively, and 0.245 µM and 9.66 µM against the Gram-positive bacteria *B. cereus* and *S. aureus*, respectively. APOC1_64–88_ showed substantial activity against *E. coli, P. aeruginosa* and *B. cereus* when compared to the performance of the known CAMP LL-37, which had EC_50_ values of 0.00821 µM, 0.525 µM and 0.0287 µM, against these bacteria, respectively. The nested peptide APOC1_67–88_ exhibited increased potency, relative to the longer peptide, with EC_50_ values of 0.151 µM, 0.948 µM, 0.210 µM and 7.08 µM against *E. coli, P. aeruginosa B. cereus*, and *S. aureus*, respectively. The performance of APOC1_67–88_ was similar to EC_50_ values obtained by LL-37. Interestingly, neither APOC1 peptide was predicted to be strongly antimicrobial by the CAMP prediction algorithms tested, again highlighting the difficulty of predicting antimicrobial peptides based on our current understanding of the factors defining peptide antimicrobial activity. Only the APD2 database correctly predicted that these peptides would have antimicrobial activity.

A1P_394–428_, a fragment of alpha-1-antiproteinase, is a 35-residue peptide with a predicted +4 charge at neutral pH. Alpha-1-antiproteinase, also known as alpha-1-antitrypsin, is a serine protease inhibitor (Serpin), and is a major protease inhibitor present in human body that has been linked to anti-inflammatory immune response [27]. Although 4/5 of the CAMP-predictor algorithms indicated it would not be antimicrobial, the peptide showed significant activity against *E. coli* (0.0986 µM), *B. cereus* (0.770 µM) and *S. aureus* (1.36 µM), and exhibited moderate activity against *P. aeruginosa* (4.35 µM). Based on its performance relative to that of LL-37 against the panel of bacteria, A1P_394–428_ appears to show good broad-spectrum antimicrobial effectiveness. Only the APD2 database correctly predicted that this peptide would have antimicrobial activity.

Two nested peptides derived from fibrinogen, FGG_398–413_ (16aa) and FGG_401–413_ (11aa) were generated, both of which carry a nominal charge of +4 at physiological pH. Fibrinogen has been associated with coagulation, which is initiated through proteolytic processing via thrombin. However, it has recently been suggested that treatment of fibrinogen with thrombin may also generate peptides that exhibit antimicrobial activity [28]. FGG_401–413_ was predicted by all but one of the algorithms to have antimicrobial activity, while only 2 out of 5 algorithms tested predicted FGG_398–413_ to be a CAMP. Interestingly, neither FGG_398–413_ nor FGG_401–413_ was found to have strong antimicrobial activity except against *E. coli* (EC_50_ = 0.33 µM and 0.245 µM, respectively). However, FGG_398–413_ proved to be moderately effective against Gram-positive *S. aureus* (EC_50_ = 2.84 µM). Against *P. aeruginosa* and *B. cereus*, FGG_398–413_ exhibited poor antimicrobial activity with EC_50_ values of 7.02 µM and 9.35 µM, respectively. The nested peptide FGG_401–413_ displayed a decrease in potency relative to FGG_398–413_ against *P. aeruginosa*, *B. cereus*, and *S. aureus* with EC_50_ values of 11.1 µM, 18.7 µM and 31.8 µM, respectively. While FGG_398–413_ presented mixed antimicrobial effectiveness when compared to the performance of LL-37, the nested peptide FGG_401–413_ showed fairly poor antimicrobial activity against the bacteria tested, except for *E. coli*. These FGG peptides potentially represent a counter-example to the APOC1 peptides, which were active but were predicted not to be, except for the APD2 database. In this case, only APD2 correctly predicted that the FGG peptides would be inactive.

Based on the antimicrobial performance data for the eight selected peptides, *E. coli* (ATCC 25922) appears to be very sensitive to CAMP activity (Fig. 4), suggesting that performance against this strain may not be a good predictor of broad-spectrum activity. This is reflected in the performance of the peptides against the three other bacteria tested, emphasizing the importance of testing peptides against multiple strains and Gram-types of bacteria for a more complete view of their potential activity.

## Discussion

In order to overcome the limitations associated with current approaches to CAMP discovery, we have developed a novel and promising method for identifying new and potentially useful antimicrobial peptides. In this process, hydrogel microparticles harvest functional CAMPs based on their physico-chemical properties. Coupled with subsequent mass spectral analysis of the intact captured peptides, this process eliminates current labor-intensive, low-yield processes associated with conventional approaches for CAMP identification. As an initial proof-of-principle, we demonstrated the ability of the hydrogel particles to harvest known CAMPs from plasma using commercial alligator plasma spiked with buforin, SMAP-29, and indolicidin. The particles were found to capture the model CAMPs from plasma and subsequently release them for analysis by mass spectrometry.

Application of the particle-based CAMP discovery process to a 100 µL sample of plasma from the American alligator resulted in the capture and sequencing of 568 alligator peptides. Evaluation of these sequences using a combination of rational analysis and web-based predictor algorithms identified 45 potential CAMPs. Eight of these peptides were synthesized and their antimicrobial effectiveness evaluated, which resulted in the discovery of five peptides (APOC1_64–88_, APOC1_67–88_, A1P_394–428_, FGG_398–413_ and FGG_401–413_) that exhibited antimicrobial activity against one or more of the Gram-positive and/or Gram-negative bacteria tested.

We have identified at least one peptide (APOC1_67–88_) with significant antimicrobial activity against the Gram-negative bacterium *P. aeruginosa* (EC_50_ = 0.948 µM), similar to the activity of the well-studied CAMP LL-37 (EC_50_ = 0.525 µM). This peptide is also able to achieve 50% killing of *B. cereus* cells at a concentration within an order of magnitude of the LL-37 EC_50_ value. We also identified A1P_394–428_ as a peptide that has significant activity against the Gram-positive bacterium *S. aureus* (EC_50_ = 1.36 µM), which is similar to that of LL-37 (EC_50_ = 0.552 µM).

The sequences of these new peptides are not derived from any of the known classes of CAMPs, but instead are fragments of larger proteins with diverse functions. The *in vivo* role of these peptides or their source proteins in innate immunity or host defense of the American alligator is not yet known, and will be the subject of further study.

The successful identification of five novel peptides that exhibit the ability to exert a direct antimicrobial effect on bacteria from 100 µL of alligator plasma substantiates the potential utility of this new CAMP-discovery process. However, the mixed performance of the prediction algorithms and databases to accurately predict the activity of the eight selected peptides exposes limitations associated the existing algorithms, and more broadly our understanding of the relationship between peptide physico-chemical properties and CAMP effectiveness. These results suggest that the data upon which the prediction algorithms [10–12] are based fail to sufficiently capture the sequence and performance diversity that exists among antimicrobial peptides.

Combined, the data from the *P. aeruginosa*, *S. aureus* and *B. cereus* bacterial assays suggest that three of the new alligator peptides have significant and potentially broad-spectrum antibacterial activity (APOC1_64–88_, APOC1_67–88_, A1P_394–428_), while two do not (FGG_398–413_ and FGG_401–413_), when compared to the human peptide LL-37. In performance assays, *E. coli* was affected by all 5 peptides with roughly the same sensitivity, suggesting that this strain is very sensitive to CAMPs, and is less able to discriminate between peptides and the scope of their effectiveness. Therefore, the following analysis focuses on their performance against *P. aeruginosa*, *S. aureus* and *B. cereus*. Calculations from the *CAMP* program [10] did not accurately predict the positive antimicrobial activity of any of the active peptides in this study. The *CAMP* program’s SVM calculation was correct in predicting the non-activity of one peptide (ASAP130LP), while *CAMP* program’s DA calculation only correctly predicted the non-activity of two peptides (ASAP130LP and NOTS_17–38_). *AntiBP2*’s prediction of activity was incorrect for each of the 6 peptides for which values were computed. The *APD2* database [12] was perhaps the most accurate, correctly predicting the activity for 5/8 peptides, and was the only database to positively predict the activity of the three active peptides (APOC1_64–88_, APOC1_67–88_, A1P_394–428_), and the inactivity of the two FGG peptides. This result may reflect the better potential predictive power of this database, built upon homology of the query peptide against a large collection of verified and active peptides from the literature. However, the *APD2* database incorrectly predicted that 3 of the inactive peptides would be active (AVTG2LP, ASAP130LP, NOTS_17–38_).

This first application of the bioprospecting approach to CAMP discovery has yielded immediate success in the form of newly identified peptides; however, the greatest benefit may lie in the insights that it has provided into how to improve the process. Enhancing CAMP harvesting selectivity and modifying chromatography and mass spectrometry parameters will allow more efficient capture and identification of CAMP candidates. Furthermore, until more reliable algorithms become available, it will be necessary to continue casting a relatively wide net when predicting whether captured peptides are potential CAMPs. We anticipate that these efforts will not only lead to the discovery of new CAMPs, but will generate a body of sequence and performance data for both CAMPs and CAMP-like peptides that can be used to develop new or refine existing CAMP-prediction algorithms in order to improve their reliability and versatility. Such developments would in turn improve the efficiency and utility of the bioprospecting CAMP discovery process.

Although this CAMP discovery process has only been used to analyze samples of alligator plasma to date, the relatively small sample volume requirement and the fact that the process is sample agnostic make it applicable to a broad spectrum of animals that were previously thought inaccessible, such as organisms of smaller body mass or endangered species. This will allow analysis of the peptidomes in some of the world’s most remarkable species, to dramatically expand the current CAMP library and potentially unlock strategies for overcoming antibiotic resistance via the discovery of new antimicrobial peptides. Beyond CAMP discovery, we envision the bioprospecting approach being applied to mining peptidomes for other classes of peptides for therapeutic and biotechnology applications.

## Materials and Methods

### Bacterial Strains

*Escherichia coli* (ATCC 25922), *Bacillus cereus* (ATCC 11778), *Pseudomonas aeruginosa* (ATCC 9027), and *Staphylococcus aureus* (ATCC 25923) used in these studies were purchased from the American Type Culture Collection (Manassas, VA). Bacteria were grown following ATCC recommended protocols for each strain and frozen aliquots were prepared in 20% glycerol and stored at -80°C for further use.

### Materials

The peptides used in these studies were custom synthesized by ChinaPeptides Company (Shanghai, China) and had purities of ≥ 95%, based on chromatographic analysis of the purified peptides. Synthetic peptides were verified on a Thermo LTQ mass spectrometer (Thermo Fisher Scientific, Waltham, MA, USA). The broad-spectrum protease inhibitor cocktail Protease cOmplete was purchased from Roche Diagnostic, Corp. (Indianapolis, IN). Resazurin, sodium salt is purchased from Sigma-Aldrich (St. Louis, MO). *N*-Isopropylacrylamide (NIPAm), *N*, *N*′-Methylenebisacrylamide (BIS), Acrylic acid (AAc), 2-Acrylamido-2-methylpropane sulfonic acid (AMPS), Methyl Acrylate (MA), Lithium hydroxide (LiOH) and potassium persulfate (KPS) are all purchased from Sigma-Aldrich (St. Louis, MO). Mueller Hinton Broth (MHB) was purchased from Becton Dickinson and Company (Sparks, MD). Phosphate buffered saline (PBS) was purchased from Corning-cellgro (Manassas, VA). Commercial alligator plasma was purchased from Bioreclamation (Westburg, NY). Alligator blood was acquired from St. Augustine’s Alligator Farm (St. Augustine, FL). All experiments involving alligators were carried out with compliance with relevant guidelines, using protocols approved by the GMU IACUC.

### Particle Synthesis

The p-NIPAm-based particles are synthesized using one-pot free radical precipitation polymerization following previously published protocols [8]. Particles incorporating AAc and AMPS are synthesized as follows: NIPAm (2.98 g, 26.28 mmol), BIS (0.111 g, 0.72 mmol), AAc (370 µL, 5.4 mmol), and AMPS (0.746 g, 3.69 mmol) are dissolved in 120 mL H_2_O. The reaction is heated to 72–78°C with stirring while degassing with N_2_. Once the reaction has stabilized at 77°C, the polymerization is initiated with the addition of KPS (24 mg, 8.88 µmol), and allowed to continue for three hours at 77°C under N_2_. The reaction is allowed to cool and the resulting particle suspension is dialyzed against water at room temperature for three days, with the dialyzed particles lyophilized and ready for use in harvesting. Core-shell particles incorporating AAc are synthesized using a similar approach, with NIPAm (1.08 g, 9.54 mmol), BIS (55.5 mg, 0.36 mmol), and MA (734 µL, 8.10 mmol) as the initial monomer feed dissolved in 60 mL H_2_O. The shell is introduced three hours after initiation, with the addition of a new combination of feed monomers, NIPAm (2.0 g, 17.64 mmol) and BIS (55.5 mg, 0.36 mmol) in 60 mL H_2_O. The reaction is allowed to continue with stirring for another 3 hours under N_2_ at 74°C. Particles are dialyzed to remove unreacted monomer and byproducts. The core-shell MA particles are saponified using lithium hydroxide in aqueous methanol to convert the MA units to AAc. The hydrated diameters of the particles are determined using dynamic light scattering at a scattering angle of 90°. The AAc/AMPS particles were determined to be 591.9 ± 78.6 nm in diameter and the core-shell AAc particles 1290 ± 214 nm. The particles are combined in a 50:50 mixture by weight for use in harvesting.

### Harvest and Elution

Alligator plasma* (100 µL) is diluted into 1.6 mL of Hydrogel particles (40 mg) suspended in aqueous 10 mM Tris-Cl for a final volume of ~1.7 mL and pH of 5. After incubating approximately 18 hours at room temperature, the plasma–particle harvest mixture is centrifuged at 16.1 × 10^3^ *rcf* to pellet the particles, and the pelleted particles are resuspended in 10 mM Tris-Cl buffer (pH 7.4). This centrifugation and resuspension process is repeated at least two times to ensure removal of excluded proteins and peptides. Following the final wash with Tris-Cl buffer, the pelleted particles are suspended in an elution solution of 1:1 trifluoroethanol (TFE): 0.1% TFA in water. The particles are gently agitated for one hour at room temperature before pelleting (as described above). The supernatant layer, containing eluted captured peptides, is set aside for later use. To ensure all peptides had been removed from the particle interior, the elution process is repeated three more times with 20 minute incubations. All elution supernatants are combined and dried via speed vacuum before de-salting by solid-phase extraction with a C_18_ Zip-Tip (Millipore, Billerica, MA, USA) for mass spectrometry analysis.

* In the case of the model plasma sample, commercial alligator plasma was spiked with the three CAMPs buforin, SMAP-29, and indolicidin (60 pmol each). For CAMP discovery, plasma from ionomycin stimulated alligator blood (1 μM, 30 minutes, 30°C) is used. Immediately following stimulation, an aliquot of protease inhibitor solution (10 µL / 100 µL of plasma), is added to the stimulated plasma.

### LC-MS/MS

Particle eluate is analyzed by high-sensitivity nanospray LC–MS/MS with an LTQ-Orbitrap Elite mass spectrometer (Thermo Fisher Scientific, Waltham, MA, USA) equipped with an EASY-nLC 1000 HPLC system (Thermo Fisher Scientific, Waltham, MA, USA). The reversed-phase LC column is a PepMap 50 μm i.d. × 15 cm long with 3 μm, 100 Å pore size, C_18_ resin (Thermo Fisher Scientific, Waltham, MA, USA). The mobile phase is a gradient prepared from 0.1% aqueous formic acid (mobile phase component A) and 0.1% formic acid in acetonitrile (mobile phase component B). After sample injection, the column is washed for 5 min with A; the peptides are eluted by using a linear gradient from 0 to 50% B over either 45 min or 2 hours and ramping to 100% B for an additional 2 min; the flow rate is 300 nL/min. The LTQ-Orbitrap Elite is operated in a data-dependent mode in which each full MS scan (120,000 resolving power) is followed by five MS/MS scans (120,000 resolving power) in which the five most abundant molecular ions are dynamically selected and fragmented by electron transfer dissociation (ETD) using fluoranthene as the electron transfer reagent. “FT master scan preview mode”, “Charge state screening”, “Monoisotopic precursor selection”, and “Charge state rejection” were enabled so that only the ≥ 3+ ions are selected and fragmented by ETD.

Tandem mass spectra were imported directly as .RAW files and analyzed by PEAKS *de novo* sequencing software version 6 (Bioinformatics Solutions Inc., Waterloo, ON Canada). PEAKS first performs a *de novo* sequence analysis using the ETD MS/MS data. Mass tolerance for precursor ions was 10 ppm and mass tolerance for fragment ions was 0.05 Da. Data were analyzed with no enzyme specificity, along with oxidation (+15.9949 Da) on methionine as a variable post translation modification. Confident *de novo* peptide identifications were achieved by filtering Average Local Confidence (ALC) to ≥ 30%. Sequence tags from the confident *de novo* sequences are searched against 2 separate databases. The first is an expressed sequence tag (EST) database obtained by searching the EST database at NCBI (http://www.ncbi.nlm.nih.gov) for all known alligator EST sequences. A total of 5469 alligator EST sequences are found from a number of sources, including the Adult American Alligator Testis Library (University of Florida, Department of Zoology, Gainesville, FL), the Juvenile American Alligator Liver Library (NIBB, Japan), and the Adult American Alligator Liver Library (University of Florida, Department of Zoology, Gainesville, FL). All were downloaded to the local computer hard drive and subsequently uploaded as a database in PEAKS. The second database was an *Alligator mississippiensis* transcriptome obtained from the International Crocodilian Genome Working Group (www.crocgenomes.org) [16]. A 1% false discovery rate (FDR) was used as a cut-off value for reporting peptide spectrum matches (PSM) from either database. Peptides of interest, both those that are sequenced from the databases and those that have only a *de novo* sequence and thus no database equivalent are all manually verified (Fig. 3). For *de novo*-only sequences, only leucine (L) was denoted since it is indistinguishable from isoleucine (I) by ETD fragmentation.

### CAMP prediction

Verified sequences were input into web-based CAMP prediction sites, *CAMP* database [10], *AntiBP2* [11] *and APD2* [12], where each peptide was scored and the likelihood of their having antimicrobial activity predicted (Table 3). Furthermore, the physico-chemical properties (length, molecular weight, nominal solution charge, pI and hydrophobicity) of all verified sequences were calculated and sorted. Length, charge and hydrophobicity were calculated using the *CAMP* database properties calculator [10]. *CAMP* database calculates hydrophobicity based on the per-residue hydrophobicity scale determined by George Rose *et al.* [29]. Molecular weight and pI were calculated using ExPASy compute MW/pI tool (http://web.expasy.org/compute_pi/).

### Antibacterial Performance

Frozen enumerated bacterial aliquots were thawed on ice and mixed. For each strain, bacteria are diluted to 2 × 10^6^ CFU/mL in sterile 10 mM sodium phosphate (pH 7.4) and added in 50 µL aliquots to the wells of a 96-well black microtiter plate (Greiner Bio-One 655201) containing 50 µL volumes of serially diluted CAMP, dissolved in the same phosphate buffer. Control wells contain bacteria with no peptide. The microtiter plate was incubated for 3 hours at 30°C (*B. cereus*) or 37°C for other strains. After three hours, 100 µL of PBS solution with dissolved resazurin and MHB was added to each well. The amount of resazurin and MHB that was added is bacterial strain dependent, with the final resazurin (µM)/ MHB (wt/vol) concentrations being 100 µM/ 0.2% (wt/vol) for *E. coli*, 12.5 µM/ 0.05% (wt/vol) for *B. cereus*, 25 µM/ 2.2% (wt/vol) for *P. aerugoinsa*, and 50 µM/ 2.2% (wt/vol) for *S. aureus*. The metabolic conversion of resazurin to resorufin is bacterial strain dependent and requires concentrations optimized for each species to achieve a high-throughput assessment. Following addition of resazurin/MHB buffer, the plate was immediately placed in either a SpectraMax Gemini EM plate-reading fluorimeter (*E. coli* and *B. cereus*) or a TeCan Safire 2 fluorimeter (*S. aureus* and *P. aeruginosa*) for incubation overnight at either 30°C (*B. cereus*) or 37°C (other strains) while monitoring fluorescence for each well.

Fluorescence data was collected from each well during the monitoring period using equations compiled by microplate data software (SoftMax Pro 4.5 or Magellen 6). Onset time of half maximal fluorescence (T_0.5_) was used for quantifying bacterial concentrations. Standard curves were generated in preliminary experiments using serially diluted bacterial suspensions (~10^6^ CFU/mL- 10^3^ CFU/mL) without CAMPs. Observed T_0.5_ values are plotted against initial CFU counts that had been determined by plating on MHB agar plates, and the relationships analyzed by linear regression, affording the following equations:

$$\text{log}({\text{CFU}}_{E.coli})=(-1.94\times 10^{-4})\left(T_{0.5}\right)+8.28\left(R^{2}=0.999\right)$$ (1)

$$\text{log}({\text{CFU}}_{B.cereus})=(-1.42\times 10^{-4})\left({\text{T}}_{0.5}\right)+5.30\left({\text{R}}^{2}=0.995\right)$$ (2)

$$\text{log}({\text{CFU}}_{P.aeruginosa})=(-1.25\times 10^{-4})\left({\text{T}}_{0.5}\right)+9.22\left({\text{R}}^{2}=0.991\right)$$ (3)

$$\text{log}({\text{CFU}}_{S.aureus})=(-9.03\times 10^{-4})\left({\text{T}}_{0.5}\right)+7.99\left({\text{R}}^{2}=0.993\right)$$ (4)

These linear regression equations are used to interpolate survival following incubation of bacteria with CAMPs, with the CFU for each well being determined based on their respective T_0.5_ values. Correlating bacterial CFU values for wells containing peptide with control wells containing no CAMPs it is possible to determine bacterial survival for wells containing CAMPs. The resazurin assay was validated with traditional dilution plating assays and the resazurin-based values were found not significantly different based on t-test and F-test statistical evaluations (see Table A and Fig. A in S1 File).

### Statistical Analysis

Antibacterial measurements are performed in triplicate. Bacterial survival results generated for each CAMP are fit to a variable-slope sigmoidal regression model to reveal bacterial survival curves using Prism 5 (GraphPad Software, Inc). Best-fit values generated for the survival curve-fit parameter log (EC_50_) are used as performance criteria. Log (EC_50_) represents the log of the peptide concentration (PC) that causes a halfway response between S_min_ and S_max_, the minimal and maximal survival values, respectively, where Hill slope (HS) is the parameter used to quantify the steepness of the transition slopes in sigmoidal survival curves.

$${\text{Bacterial Survival = S}}_{\text{min}}\text{+}\frac{{\text{S}}_{\text{max}}{\text{-S}}_{\text{min}}}{{\text{(1+10}}^{{\text{((log(EC}}_{\text{50}}\text{))-((log(PC))}*\text{HS))}})}$$ (5)

Antilogs of the log (EC_50_) values, the EC_50_ values, are tabulated and 95% confidence intervals (CI) are presented to demonstrate overlap and statistical significance. This data is presented in Table 2 and in graphical format in Fig. 4.

## Supporting Information

S1 File This file contains Table A and Figure A. Table A, Comparison of Antibacterial Performance EC_50_ and Hill Slope Values Determined by Dilution Plating and by Resazurin Assays. EC_50_ and Hill slope values for LL-37 against *E. coli* and *B. cereus* with corresponding 95% confidence interval range. Figure A, Comparison of Antibacterial Performance Determined by Dilution Plating and Resazurin Assays. Antibacterial effectiveness of LL-37 against *E. coli* (A) and *B. cereus* (B) comparing results from dilution plating (represented in open circles = ○) and resazurin assays (validation data represented in open squares = □; reported manuscript data represented in closed triangles = ▲). Data were fit to equation 5, a standard variable slope dose-response equation, in order to obtain EC_50_ and Hill slope values.
(DOCX)
