# Supplementary material for: Bioprospecting the American Alligator (Alligator mississippiensis) Host Defense Peptidome
Source: PLoS One. 2015 Feb 11;10(2):e0117394. doi: 10.1371/journal.pone.0117394 (PMC4324634; doi:10.1371/journal.pone.0117394)
Supplement: S1 File — Table A, Comparison of Antibacterial Performance EC50 and Hill Slope Values Determined by Dilution Plating and by Resazurin Assays. EC50 and Hill slope values for LL-37 against E. coli and B. cereus with corresponding 95% confidence interval range. Figure A, Comparison of Antibacterial Performance Determined by Dilution Plating and Resazurin Assays. Antibacterial effectiveness of LL-37 against E. coli (A) and B. cereus (B) comparing results from dilution plating (represented in open circles = ○) and resazurin assays (validation data represented in open squares = □; reported manuscript data represented in closed triangles = ▲). Data were fit to equation 5, a standard variable slope dose-response equation, in order to obtain EC50 and Hill slope values. (DOCX) [file pone.0117394.s001.docx]

**Table A: Comparison of Antibacterial Performance EC_50_ and Hill Slope Values Determined by Dilution Plating and by Resazurin Assays.**

EC_50_ and Hill slope values for LL-37 against *E. coli* and *B. cereus* with corresponding 95% confidence interval range.


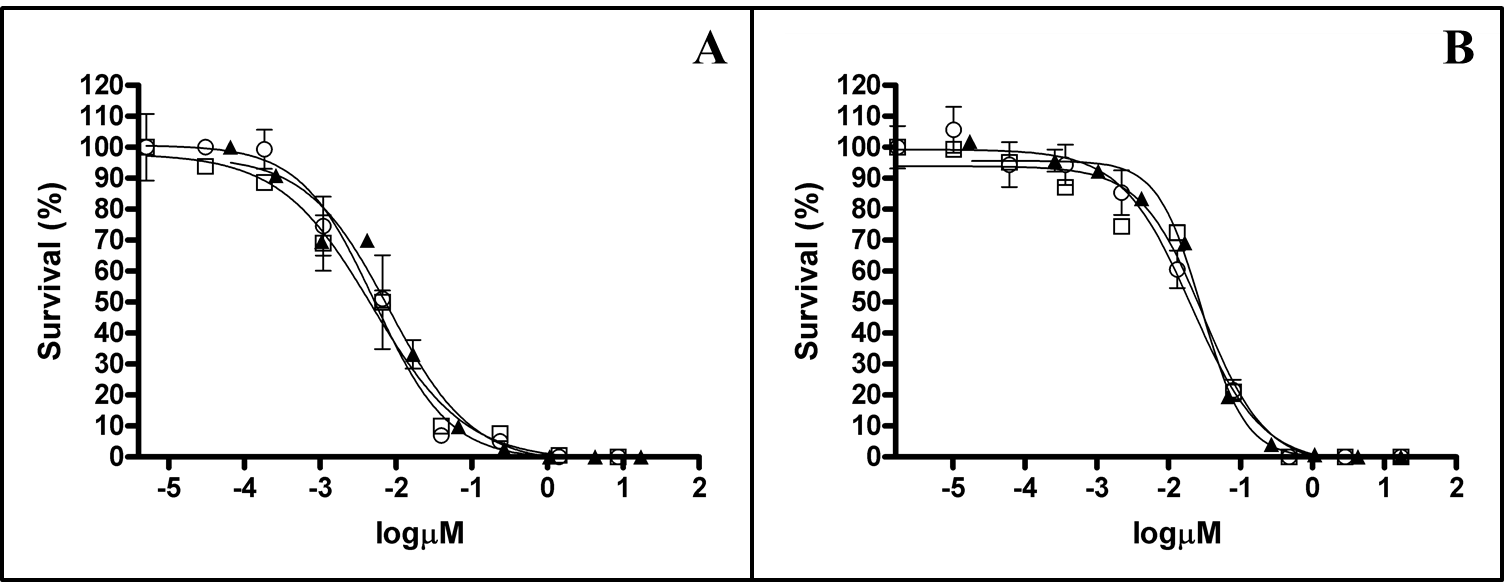


**Figure A: Comparison of Antibacterial Performance Determined by Dilution Plating and Resazurin Assays**. Antibacterial effectiveness of LL-37 against *E. coli* **(A)** and *B. cereus* **(B)** comparing results from dilution plating (represented in open circles = ○) and resazurin assays (validation data represented in open squares = □; reported manuscript data represented in closed triangles = ▲). Data were fit to equation 5, a standard variable slope dose-response equation, in order to obtain EC_50_ and Hill slope values.

**Validation Experimental Methods:**

Assays for both *E. coli* and *B. cereus* were prepared in sextuplet from frozen enumerated cell stocks using varied concentrations of LL-37 and the procedures described in Materials and Methods. Following incubation for 3 hours at either 37° C (*E. coli*) or 30° C (*B. cereus*), three of the six assay replicates for each bacterial strain were subjected to dilution plating (onto MHB agar), while the remaining three replicates were charged with buffer containing resazurin and the viable CFU concentration for each well determined fluorometrically as described in Materials and Methods. For both the resazurin and dilution plating approaches, percent survival was established for wells containing peptide relative to control wells containing no peptide. Bacterial survival data and associated LL-37 concentrations from resazurin and plating were then fit to a variable-slope sigmoidal regression model (equation 5) using Graphpad Prism 5 (GraphPad Software, Inc.), affording the dose-response curves shown in Figure A.

Results obtained using the resazurin method were compared to those from traditional dilution plating (Figure A and Table A). Furthermore, these results were compared to those reported in the manuscript to provide additional verification of results obtained using the resazurin-based performance assay (Figure A and Table A). Best-fit log (EC_50_) and Hill slope values were used as assessment criteria for determining the extent of concordance between assay results. Here, log (EC_50_) represents the log of the concentration of LL-37 required to kill half of the bacterial population, while the Hill slope corresponds to the steepness of the transition slopes of the survival curves. Note that log (EC_50_) values were used here instead of EC_50_ values due to the fact that standard error could only be computed symmetrically using a log scale. Statistical analyses (F-test and t-test) of the log (EC_50_) and Hill slope values reported in the manuscript and those generated for the validation experiments via the resazurin assay and dilution plating methods revealed that the values were not significantly different (p ≤ 0.05 in all cases).
